# Deep-learning-based blood pressure estimation using multi channel photoplethysmogram and finger pressure with attention mechanism

**DOI:** 10.1038/s41598-023-36068-6

**Published:** 2023-06-08

**Authors:** Jehyun Kyung, Joon-Young Yang, Jeong-Hwan Choi, Joon-Hyuk Chang, Sangkon Bae, Jinwoo Choi, Younho Kim

**Affiliations:** 1 Department of Electronic Engineering, Hanyang University, Seoul, 04763 Republic of Korea; 2 SAIT, Samsung Electronics, Advanced Sensor Lab, Suwon-si, Gyeonggi-do 16677 Republic of Korea

**Keywords:** Health care, Biomedical engineering

## Abstract

Recently, several studies have proposed methods for measuring cuffless blood pressure (BP) using finger photoplethysmogram (PPG) signals. This study presents a new BP estimation system that measures PPG signals under progressive finger pressure, making the system relatively robust to errors caused by finger position when using the cuffless oscillometric method. To reduce errors caused by finger position, we developed a sensor that can simultaneously measure multi-channel PPG and force signals in a wide field of view (FOV). We propose a deep-learning-based algorithm that can learn to focus on the optimal PPG channel from multi channel PPG using an attention mechanism. The errors (ME ± STD) of the proposed multi channel system were 0.43±9.35 mmHg and 0.21 ± 7.72 mmHg for SBP and DBP, respectively. Through extensive experiments, we found a significant performance difference depending on the location of the PPG measurement in the BP estimation system using finger pressure.

## Introduction

The most accurate blood pressure (BP) measurement method involves a medical catheter^1^, wherein the BP is measured directly by inserting a catheter into an artery. This method is suitable for the long-term observation of BP changes in patients admitted to the intensive care unit (ICU). However, there is a risk of infection owing to its invasive nature^2^. Non-invasive BP measurement methods include cuff-based^3^ and cuffless methods. Cuff-based BP measurement methods use an electronic sphygmomanometer device and are widely accepted as the gold standard because they can achieve relatively high accuracy. Moreover, users can easily measure BP at home without the help of medical staff^4^. However, there are disadvantages in that the user feels uncomfortable because of the applied pressure. Furthermore, an electronic sphygmomanometer device is not easily portable.

Recently, several studies have proposed cuffless BP measurement methods^5,6^, in which the BP is predicted using various biomedical signals, such as photoplethysmogram (PPG) and electrocardiogram (ECG). The pulse transit time (PTT) or pulse arrival time (PAT) can be calculated using simultaneously measured PPG and ECG signals^5^. They are calculated using the time difference between the peaks of the two signals measured by the sensor at two different points in the artery. Although several studies have used the correlation between PTT or PAT and BP^6,7^ for BP prediction, they are not suitable for mobile devices such as smartphones or smart watches because they require two sensors in different locations to measure both PPG and ECG signals. One method for overcoming this disadvantage is to predict the BP using pulse wave analysis (PWA) from PPG signals. A PPG signal is a periodic pulse-wave signal that is correlated with the cardiovascular system. Therefore, some researchers have extracted engineered features such as height and width within the pulse wave and used them to predict BP^8^. However, extracting accurate engineered characteristics is challenging because the characteristics of the cardiovascular system differ from person to person owing to factors, such as age, disease, and drugs administered^9^.

Recently, Mukkamala *et al*. proposed the measurement of BP on a smartphone using the finger pressure method^10^, which estimates BP by using the change in the PPG envelope during blood vessel constriction caused by a gradual increase in finger pressure on the PPG sensor. However, because the sensor uses a single-channel PPG, the accuracy of BP prediction can be significantly affected by the position of the finger on PPG sensor.

In this study, we propose a novel approach to cuffless BP estimation. The contributions of this study comprise two main aspects. First, we developed a sensor that can acquire multi-channel PPG signals with different wavelengths using a finger pressure method similar to that used by Mukkamala *et al.*^10^. The proposed PPG sensor simultaneously measures multi-channel PPG signals and a finger pressure signal, while the subject’s fingertip gradually pressurizes the sensor for 40 s. The measured multi-channel PPG and finger pressure signals represent progressive pressure characteristics and have characteristics similar to those of oscillometric wave (OMW) signals and cuff pressure signals used in cuff-based BP measurement methods. Moreover, the multi-channel extension in PPG signal measurement is expected to alleviate the variation in the position of the finger on the PPG sensor. Second, we proposed a deep-learning-based BP estimation system using multi-channel PPG and finger pressure signals. The proposed deep learning-based BP prediction system consists of two parts. First, a convolutional neural network (CNN)-based model was designed, which extracts channel-specific features for BP estimation from the multi channel PPG and finger pressure signals. Second, a multi-channel attention network for improved BP estimation accuracy was proposed, which combined the latent features obtained from the single-channel BP estimators to produce a new attention-weighted feature. The combined feature was subsequently used for the final BP estimation.

## Description of sensors and datasets

The oscillometric method for measuring BP uses the changes in PPG amplitudes caused by the occlusion of blood vessels. To accurately measure PPG signals at high signal-to-noise ratios, the optical path of the sensor must include blood vessels. However, because fingers have a complex vascular structure, there are small arteries on both sides of the finger and across the tip of the nail. Therefore, it is difficult to accurately form an optical path that includes blood vessels using a single-channel PPG sensor. To overcome this challenge, we developed a multi-channel PPG sensor with a wide field-of-view (FOV).

### Sensors

**Figure 1:**
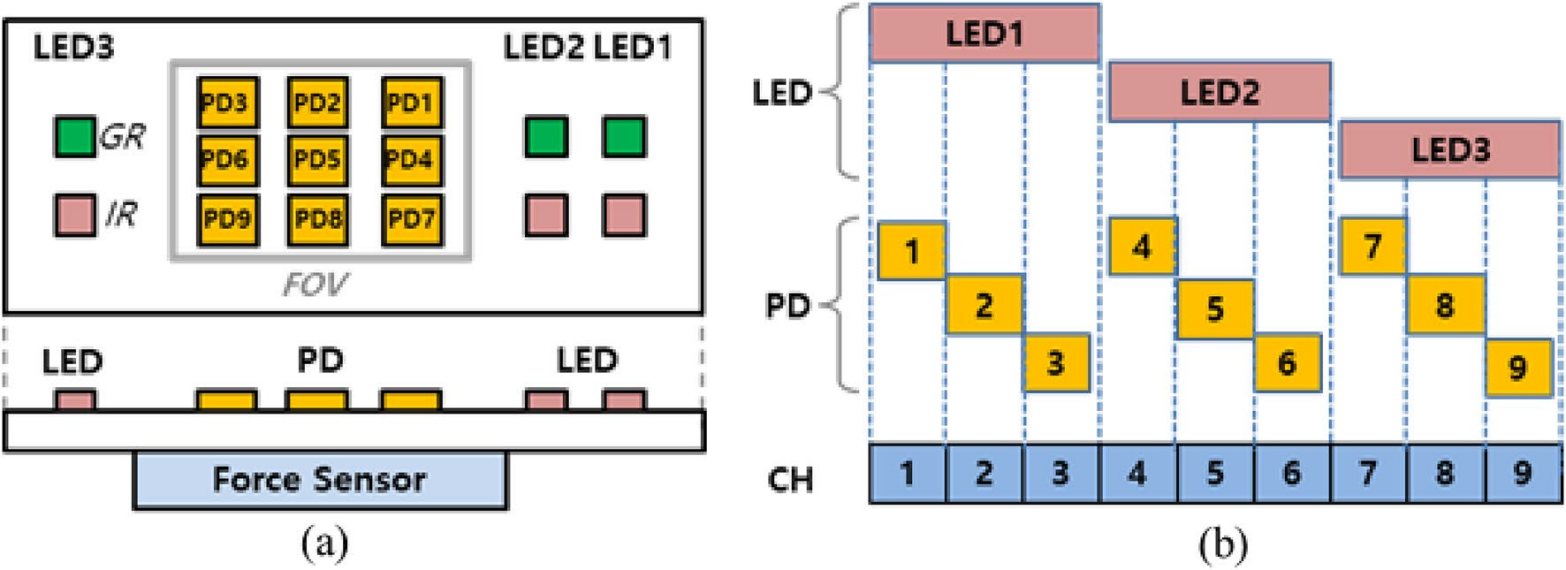
Structure of multi-channel PPG sensor: (**a**) structure of LED-PD and Force sensor and (**b**) operating time of multi-channel PPG.

**Figure 2:**
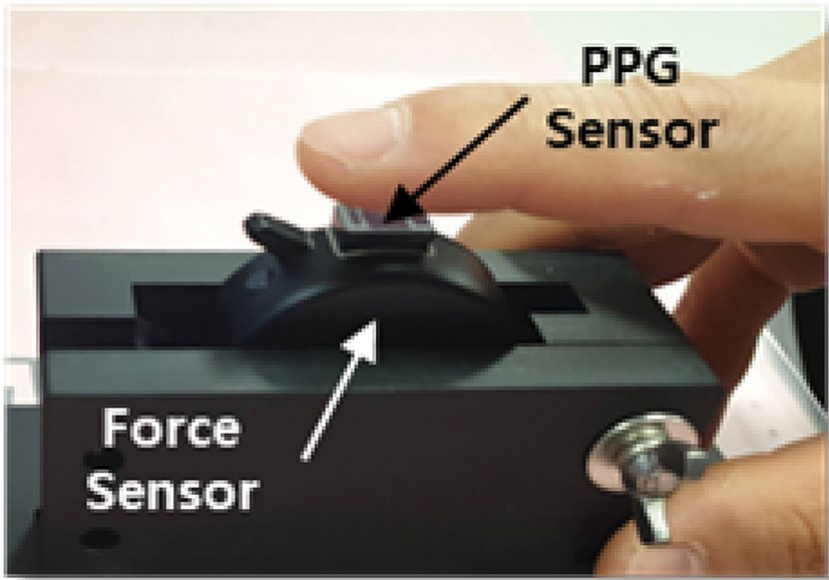
Experimental setup of the proposed sensor.

The proposed PPG sensor comprises the following components, as shown in Fig. 1a: three green and three infra-red (IR) light emitting diodes (LEDs) (wavelengths of 535 nm and 850 nm, respectively) and nine photodetectors (PDs). The LED and PD for multi channel PPG operate according to the timing chart, as shown in Fig. 1b: three LEDs for row positions (top, middle, and bottom PDs), and three PDs for column positions (right, center, and left PDs). LEDs were placed on both sides of the PDs to account for the finger blood vessel structure. In addition, the FOV for the multi channel was 5 mm $\times$ 4.5 mm, and the overall size was 12 mm $\times$ 7.5 mm, enabling a finger to cover the entire sensor. We used this sensor to measure 9-channel PPG signals from an IR LED with a sampling rate of 43 Hz. Figure 2 shows the system setup for the experiment. The PPG sensor was configured as a button on the experimental support, and the commercial force sensor was located under the PPG sensor to detect the force signal exerted by the finger. Notably, the 9-channel PPG and force signals were synchronized, measured, and subsequently fed into the analog-to-digital converter of the built-in mainboard.

### Data collection

Clinical trials were conducted separately at two different sites: the MONIKI Hospital in Russia and Samsung Medical Center in Korea. Dataset1 was collected at the MONIKI Hospital and contained 1,450 cases from 290 participants; it was used to train the proposed BP estimation system. Dataset2 was collected at the Samsung Medical Center and contained 865 cases from 186 participants; it was used for training and testing. Each case included 40s synchronized 9-channel PPG and force signals. And reference BP obtained from two medical staff using auscultation was also included. Data collection was approved by the ethical committee of Samsung Medical Center (IRB Protocol No: 2020-06-065). The study design followed the International Standard (ISO 81060-2)^11^ which was describing relevant guidelines for clinical investigation of Non-invasive Sphygmomanometers including subject requirements (minimum of 85 subjects and 255 BP values), reference readings (mean value from 2 observers using double stethoscope). All examinees provided informed consent before the measurements were conducted.

For clinical trials, the proposed multi-channel PPG system was used in compliance with standard protocols (ISO 81060-2). The reference BP was measured by two medical staff members using the auscultation method, and five measurements were conducted for each subject. The participants took a break of at least 5 min between measurements to ensure stability. The finger was then placed on a pre-marked guideline. After the measurement started, they were asked to gradually press the sensor with their index finger for 40 s while watching the pressure increase guide displayed on the computer screen. Table 1 lists the demographic information of the dataset.

**Table 1:** Demographic information in the data.

	Dataset1		Dataset2		Total	
No. of subjects	290		186		476	
No. of cases	1450		865		2315	
Ref. SBP [mmHg]	121.74±19.26		116.61±18.38		119.83±19.09	
	Min	Max	Min	Max	Min	Max
	82.5	195.0	80.0	185.0	80.0	195.0
Ref. DBP [mmHg]	78.52±12.47		75.93±12.98		77.55±12.73	
	Min	Max	Min	Max	Min	Max
	49.0	129.5	46.0	110.0	46.0	129.5
Age [years]	41.47±14.69		36.71±10.49		39.61±13.41	
	Min	Max	Min	Max	Min	Max
	17	69	20	64	17	69
Height [cm]	170.49±8.95		166.35±8.47		168.87±9.0	
	Min	Max	Min	Max	Min	Max
	150.0	195.0	147.7	185.1	147.7	195.0
Weight [kg]	78.31±19.03		66.83±14.89		73.82±18.40	
	Min	Max	Min	Max	Min	Max
	46.0	148.0	41.8	111.1	41.8	148.0

## Results

### Setup

We used dataset1 of 290 participants and dataset2 of 186 participants for the training, validation, and testing of the BP estimation system. We divided the training, validation, and test data such that there were no overlapping participants. Dataset1 was used only for training and validation, whereas dataset2 was used only for training and testing. we split dataset1 with 183 participants for training and 107 participants for model validation. In addition, dataset2 was divided into file-folds without overlapping participants, one-fold was used for testing and the remaining folds were used for training, and each of the five-folds was tested in turn. Therefore, we performed model verification through a five-fold cross-validation of dataset2. All the five-fold BP estimation results obtained using dataset2 are presented in.

To train the nine single-channel CNN-based feature extractors and multi channel attention mechanism, the Adam optimizer^12^ was used with $\beta _1=0.9$ and $\beta _2=0.999$, a learning rate of 0.005, and a mini-batch size of 64. To improve the generalization of the proposed BP estimation system, a $\ell _2$ regularization term with a scale of 0.005 and dropout rate of 0.3 were used. Detailed model parameters are summarized in Table 2.

**Table 2:** The detailed parameters of the model.

Method	Input shape	Layer	Kernel/Stride/Out	Output shape
CNN-based feature extractor	1720 × 3 1720 × 3 215 × 1	Conv1D + BN + ReLU	7/2/4	860 × 4 860 × 4 108 × 4
	860 × 4 860 × 4 108 × 4	Max pooling	7/1/-	860 × 4 860 × 4 108 × 4
	860 × 4 860 × 4 108 × 4	Conv1D + BN + ReLU + Dropout	3/1/8	860 × 8 860 × 8 108 × 8
	860 × 8 860 × 8 108 × 8	Conv1D + BN + Shortcut Sum + ReLU	3/2/8	430 × 8 430 × 8 54 × 8
	430 × 8 430 × 8 54 × 8	Conv1D + BN + ReLU + Dropout	3/1/16	430 × 16 430 × 16 54 × 16
	430 × 16 430 × 16 54 × 16	Conv1D + BN + Shortcut Sum + ReLU	3/2/16	215 × 16 215 × 16 27 × 16
	215 × 16 215 × 16 27 × 16	Conv1D + BN + ReLU + Dropout	3/1/32	215 × 32 215 × 32 27 × 32
	215 × 32 215 × 32 27 × 32	Conv1D + BN + Shortcut Sum + ReLU	3/2/32	108 × 32 108 × 32 14 × 32
	108 × 32 108 × 32 14 × 32	Average pooling	-/-/-	1 × 32 1 × 32 1 × 32
	1 × 32 1 × 32 1 × 32	Concatenate	-/-/-	1 × 96
	1 × 96	FC + sigmoid	-/-/16	16 × 1
Attention mechanism	16 × 9(Z)	Fully connected layer	-/-/1	1 × 9(W)
	1 × 9(W)	Softmax	-/-/-	1 × 9(W)
	16 × 9(Z) 1 × 9(W)	Multiply	-/-/-	16 × 9
	16 × 9	Summation	-/-/-	16 × 1
	16 × 1	Output layer	-/-/1	1 × 1

**Table 3:** Comparison of SBP performance according to single-channel PPG signal. significant values are in bold.

Method	PPG channel	SBP (mmHg)					
		Mean error ± standard deviation (correlation coefficient)					
		Fold 1	Fold 2	Fold 3	Fold 4	Fold 5	Average
CNN-based single-channel SBP estimation	Ch 1	− 3.09±10.58	3.81±11.66	− 0.61±10.03	− 0.48±8.78	− 2.63±8.63	− 0.65±10.26 (0.83)
	**Ch 2**	− **1**.**36**±**10**.**37**	**4**.**46**±**11**.**0**	**3**.**01**±**9**.**56**	**1**.**66**±**8**.**86**	**1**.**91**±**8**.**9**	**1**.**9**±**9**.**94** **(0.84)**
	Ch 3	− 2.03±12.64	4.89±11.83	0.95±10.25	1.25±8.5	3.94±9.4	1.79±10.89 (0.81)
	Ch 4	2.09±11.12	1.24±11.73	1.68±10.17	2.95±9.02	1.87±8.7	1.98±10.2 (0.83)
	Ch 5	1.44±11.24	1.73±12.0	1.25±9.42	1.15±9.11	0.34±8.87	1.17±10.19 (0.83)
	Ch 6	3.74±11.43	2.88±11.72	1.83±9.95	− 1.17±8.06	0.22±8.56	1.46±10.18 (0.83)
	Ch 7	1.44±11.95	3.99±12.16	3.65±10.63	3.94±9.35	− 2.75±9.07	2.01±10.99 (0.8)
	Ch 8	4.34±10.93	2.79±11.87	2.73±10.28	1.65±9.48	2.55±9.04	2.8±10.38 (0.83)
	Ch 9	4.25±12.6	1.54±10.88	2.19±9.08	1.44±8.27	4.47±8.92	2.79±10.15 (0.83)

**Table 4:** Comparison of DBP performance according to single-channel PPG signal. significant values are in bold.

Method	PPG channel	DBP (mmHg)					
		Mean error ± standard deviation (correlation coefficient)					
		Fold 1	Fold 2	Fold 3	Fold 4	Fold 5	Average
CNN-based single-channel DBP estimation	Ch 1	− 0.59±8.14	3.18±8.5	0.04±8.11	1.42±8.22	− 1.74±8.34	1.47±8.32 (0.74)
	Ch 2	2.41±8.21	− 2.47±8.43	1.78±8.3	2.11±8.29	1.12±8.2	2.42±8.36 (0.72)
	**Ch 3**	**0**.**14**±**7**.**94**	− **1**.**05**±**8**.**14**	**2**.**31**±**8**.**37**	**1**.**32**±**8**.**11**	− **3**.**12**±**8**.**24**	**0**.**75**±**8**.**10** **(0.76)**
	Ch 4	1.17±8.28	1.92±8.32	− 0.84±8.31	1.03±8.24	0.46±8.41	1.22±8.31 (0.74)
	Ch 5	1.04±8.19	1.26±8.28	0.47±8.24	− 2.7±8.53	− 0.07±8.1	− 0.87±8.23 (0.75)
	Ch 6	− 1.31±8.30	2.98±8.47	0.12±8.1	1.47±8.34	1.34±8.57	2.11±8.41 (0.73)
	Ch 7	2.07±8.08	3.04±8.43	− 3.21±8.37	1.13±8.57	3.19±8.52	2.41±8.39 (0.73)
	Ch 8	1.34±8.32	1.14±8.19	0.96±8.24	0.47±8.21	2.13±8.22	1.52±8.2 (0.74)
	Ch 9	0.57±8.17	0.3±8.08	1.67±8.21	3.19±8.43	1.67±8.36	1.23±8.14 (0.75)

### Evaluation metrics

We used the mean of the error (ME), standard deviation of the error (STD), and Pearson’s correlation coefficient (r) as the evaluation metrics for BP prediction. In addition to evaluating the overall BP estimation system, the BP estimation performance of the CNN-based feature extraction model for a single PPG and attention mechanism were compared and analyzed.

### Validation of the single-channel BP estimation system

Most studies that have developed a BP prediction model use Physionet’s multi-parameter intelligent monitoring in intensive care (MIMIC) online waveform database^13^ or the University of Queensland Vital Signs dataset^14^. These public datasets contain unpressurized single-channel PPG and ECG signals. Although studies^15,16^ have used self-made datasets, these usually contain unpressurized single-channel PPG signals or ECG signals. In this study, we designed a BP prediction system using the signals obtained from the proposed multi channel PPG and finger pressure sensors. Because our self-made dataset contains multi-channel PPG signals applied under pressure and finger pressure signals, a direct comparison with other BP estimation studies is difficult. Therefore, we analyzed the proposed multi-channel PPG-based BP estimation system and its components.

Tables 3 and 4 compare the SBP and DBP estimation performances for each of the nine PPG channels. In terms of the STD metric, the SBP and DBP estimation performances were the best when using the PPG signals of the 2nd and 3rd channels, respectively. In comparison, when the 7th and 6th channel PPG signals were used, the SBP and DBP estimation performances were the worst. There was a relative performance gap of approximately 9.6% in the SBP performance between the 2nd and 7th channels, and the DBP performance had a difference of approximately 3.7% between the 3rd and 6th channels. Although the multi-channel PPG signals were acquired simultaneously, the significant difference in BP prediction performance between the different channels could be attributed to the difference in the position of the finger placed on the PPG sensor for each user and the characteristic difference of fingers. Therefore, it can be stated that it is difficult to collect PPG signals consistently for all users through a single-channel PPG sensor.

### Validation of the attention mechanism system

Table 5 compares the SBP and DBP estimation performances of one of the single-channel BP estimators and the proposed multi channel attention-based BP estimator. By combining multi channel features using the multi channel attention mechanism, the SBP estimation performance improved significantly compared to its best single-channel counterpart. More specifically, the SBP estimation performances of the single-channel and multi channel systems were 9.94 and 9.35, respectively, exhibiting a 6% relative improvement. As shown in Table 5, the DBP prediction performance also improved by 4.7% when the attention mechanism method using the multi-channel PPG signal was used. Furthermore, for both SBP and DBP estimation tasks, the Pearson correlation coefficient values improved five-fold. From the data acquisition point of view, 9 channels are used for data collection, but the sampling rate is 43 Hz, which is only a minor increase and can be handled sufficiently. On the other hand, in terms of performance, if the channel is selected incorrectly, a large error can occur (e.g., the SDE of Ch 7 is 10.99, showing a difference of 1.64 mmHg). Therefore, this algorithm is effectively shown to reduce the amount of error change due to channel selection and results suggest that the proposed attention mechanism that uses multi channel PPG signals is effective.

**Table 5:** Comparison of SBP, DBP estimation performance using single-channel PPG signal and multi-channel PPG signals in attention mechanism. significant values are in bold.

Method	Mean error ± standard deviation (correlation coefficient)					
	Fold 1	Fold 2	Fold 3	Fold 4	Fold 5	Average
SBP (mmHg)						
Single-channel (Ch 2) SBP estimation	− 1.36±10.37 (0.84)	4.46±11.0 (0.88)	3.01±9.56 (0.87)	1.66±8.86 (0.82)	1.91±8.9 (0.73)	1.9±9.94 (0.84)
+ attention mechanism (Proposed)	**0**.**44**±**10**.**48** **(0.85)**	**2**.**93**±**10**.**35** **(0.9)**	**1**.**07**±**8**.**76** **(0.89)**	− **0**.**23**±**7**.**88** **(0.85)**	− **1**.**83**±**8**.**39** **(0.76)**	**0**.**43**±**9**.**35** **(0.86)**
DBP (mmHg)						
Single-channel (Ch 3) DBP estimation	0.14±7.94 (0.79)	− 1.05±8.14 (0.76)	2.31±8.37 (0.71)	1.32±8.11 (0.76)	− 3.12±8.24 (0.74)	0.75±8.1 (0.76)
+ attention mechanism (Proposed)	**0**.**73**±**7**.**63** **(0.81)**	− **2**.**3**±**7**.**97** **(0.78)**	**1**.**32**±**7**.**76** **(0.8)**	**0**.**23**±**7**.**7** **(0.8)**	− **1**.**2**±**7**.**92** **(0.79)**	**0**.**21**±**7**.**72** **(0.8)**

### Analysis of attention weights

**Figure 3:**
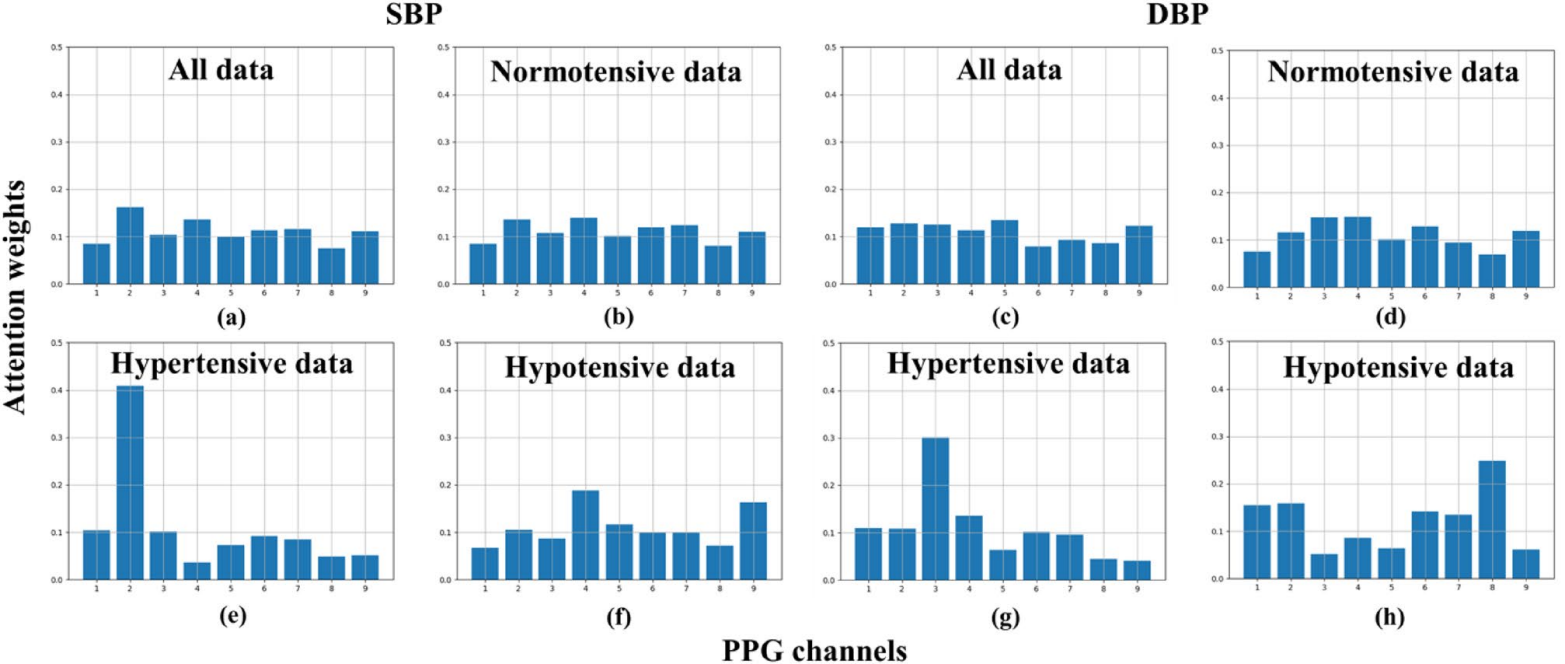
This figure is a bar graph of attention weight. In each of the 8 bar graphs, the x-axis represents 9 channels, and the y-axis represents the probability values for the importance of the channels. It is divided into hypertensive, hypotensive, and normotensive data, and is presented by SBP, and DBP.

The attention mechanism of our proposed BP estimation system is important for improving the BP estimation performance. Figure 3 shows the attention weights of hypertension, hypotension, and normotensive data obtained for the SBP and DBP estimation tasks. The average value of the attention weights of each BP group was obtained and displayed as a bar graph. Interestingly, the attention weights of some specific channels were relatively larger than those of others in both the hypertension and hypotension data. In the SBP attention mechanism, hypertension and hypotension data exhibited large attention weights in the 2nd and 4th channels, respectively. Moreover, the channel with the largest attention weight in the hypertension data tended to have a relatively low attention weight in the hypotension data, and vice-versa. These trends were also observed for the attention weights of the DBP attention mechanism. Meanwhile, the attention weight of normotensive data revealed relatively evenly distributed attention weights for both SBP and DBP estimation tasks. These results suggest that our proposed multi channel PPG sensor with a multi channel attention mechanism can be effectively used to differentiate hypertensive and hypotensive users, thereby leading to improvements overproving superior to single-channel PPG-based BP estimation models.

### Validation of the effectiveness of the proposed attention mechanism by changing the attention method

We verified that the proposed multi-channel attention mechanism could improve the BP prediction performance of single-channel models. The attention mechanism could predict BP more accurately by considering the importance of latent features extracted from multiple single-channel PPG signals. In this subsection, we confirm the effectiveness of the attention mechanism by changing its attention method. Table 6 compares the SBP and DBP performance when using part of the nine channel features instead of all of them. In the learned attention mechanism, the top two and three best single-channel systems for the validation set performance were selected and applied to the test dataset. As shown in the table, although the average attention weights indicate the relative importance of the PPG channels, the hard selection of the two or three PPG channels with the largest attention weight values was not effective. In comparison, the proposed 9-channel attention mechanism significantly improves the hard-channel selection methods in Folds 2, 3, and 4. This indicates that by using the proposed attention mechanism, the adaptive combination of multi channel PPG signals per subject is effective.

**Table 6:** Performance comparison according to the change of the attention mechanism method. significant values are in bold.

Method	Mean Error ± Standard Deviation (Correlation coefficient)					
	Fold 1	Fold 2	Fold 3	Fold 4	Fold 5	Average
SBP (mmHg)						
Only use the 2 highest attention weights	1.4±10.5 (0.83)	3.16±11.31 (0.87)	2.35±9.72 (0.85)	2.22±8.93 (0.81)	2.89±8.57 (0.73)	1.92±10.1 (0.83)
Only use the 3 highest attention weights	0.93±10.42 (0.85)	1.2±10.82 (0.89)	2.92±9.53 (0.87)	1.63±8.53 (0.83)	1.37±8.42 (0.75)	0.72±9.63 (0.85)
Attention mechanism (Proposed)	**0**.**44**±**10**.**48** **(0.85)**	**2**.**90**±**10**.**35** **(0.9)**	**1**.**07**±**8**.**76** **(0.89)**	− **0**.**23**±**7**.**88** **(0.85)**	− **1**.**83**±**8**.**39** **(0.76)**	**0**.**43**±**9**.**35** **(0.86)**
DBP (mmHg)						
Only use the 2 highest attention weights	− 2.4±7.84 (0.77)	− 1.3±8.13 (0.75)	1.34±8.1 (0.75)	0.52±7.86 (0.79)	− 2.11±8.28 (0.74)	0.53±8.04 (0.76)
Only use the 3 highest attention weights	1.3±7.71 (0.79)	− 1.7±7.99 (0.78)	0.77±7.85 (0.78)	1.21±7.72 (0.79)	− 1.18±8.09 (0.75)	0.18±7.87 (0.79)
Attention mechanism (Proposed)	**0**.**73**±**7**.**63** **(0.81)**	− **2**.**3**±**7**.**97** **(0.78)**	**1**.**32**±**7**.**76** **(0.8)**	**0**.**23**±**7**.**7** **(0.8)**	− **1**.**2**±**7**.**92** **(0.79)**	**0**.**21**±**7**.**72** **(0.8)**

**Table 7:** Comparison of SBP and DBP performance according to input signal combination in single-channel BP estimation model. **E** represents the input signal concatenated with envelopes of PPG, PPG’, and PPG” on the channel axis. **P** represents the input signal concatenated with PPG, PPG’, and PPG” on the channel axis. **F** represents the finger pressure signal. significant values are in bold.

Method	Mean error ± standard deviation (correlation coefficient)					
	Fold 1	Fold 2	Fold 3	Fold 4	Fold 5	Average
SBP (mmHg)						
E+F	0.89±13.1	2.78±12.53	0.88±11.32	1.24±9.63	2.77±9.87	1.72±11.29 (0.76)
P+F	0.15±12.82	− 0.3±12.51	1.54±10.82	0.02±9.51	− 1.81±9.66	− 0.08±11.06 (0.79)
P+E+F	− **1**.**36**±**10**.**37**	**4**.**46**±**11**.**0**	**3**.**01**±**9**.**56**	**1**.**66**±**8**.**86**	**1**.**91**±**8**.**9**	**1**.**9**±**9**.**94** **(0.84)**
DBP (mmHg)						
E+F	1.32±8.57	0.15±8.82	2.11±8.43	− 1.73±8.66	0.08±8.62	0.39±8.62 (0.69)
P+F	− 0.82±8.42	2.18±8.66	− 1.13±8.46	0.31±8.61	3.31±8.59	0.77±8.55 (0.71)
P+E+F	**0**.**14**±**7**.**94**	− **1**.**05**±**8**.**14**	**2**.**31**±**8**.**37**	**1**.**32**±**8**.**11**	− **3**.**12**±**8**.**24**	**0**.**75**±**8**.**10** **(0.76)**

### BP estimation accuracy analysis according to input signal combination

We compared the BP estimation accuracy according to the input signal combination in the single-channel BP estimation model for SBP and DBP, respectively. We compared the performance when only PPG, first and second differential signals, and finger pressure signals were used, the performance when envelope signals and finger pressure signals were used, and the performance when both were used. As shown in Table 7, in the SBP model, channel two was tested, and in the DBP model, channel three was tested. Performance was better for PPG signals than for envelope signals, and the best performance was obtained when all signals were used.

### Analysis of scatter plot and Bland–Altman plot

**Figure 4:**
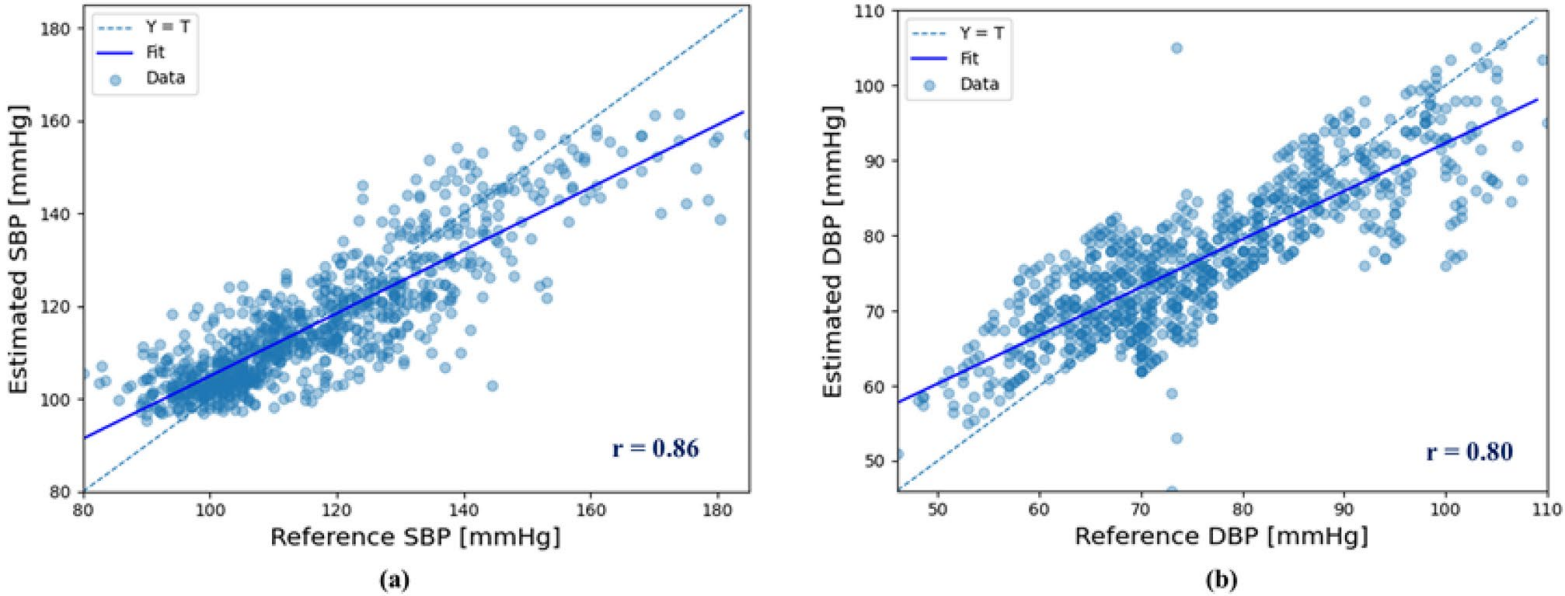
Scatter plot graph for (**a**) SBP and (**b**) DBP estimation.

**Figure 5:**
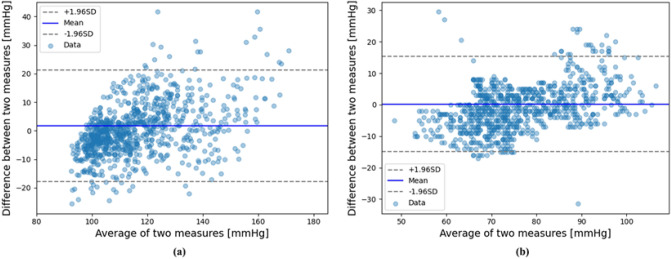
Bland–Altman plot for (**a**) SBP and (**b**) DBP estimation.

To further verify the proposed BP estimation system, the scatter plot and Bland–Altman plot^17^ for SBP and DBP estimation are shown in Figs. 4 and 5 , respectively. As shown in Fig. 4, the proposed BP prediction system showed high Pearson's correlation coefficients of 0.86 and 0.8 for SBP and DBP, respectively. The Bland–Altman plot showed that most of the SBP and DBP data samples were within the limits of agreement.

## Discussion

In this study, we developed a multi-channel PPG sensor that senses multi-channel PPG signals and finger pressure signals and proposed a cuffless BP estimation system. The acquired multi-channel PPG signals were obtained by placing a finger on the proposed sensor and gradually applying pressure. Using the developed sensor, dataset1 and dataset2 were collected from the MONIKI Hospital in Russia and the Samsung Medical Center in Korea, respectively. Dataset1 and dataset2, which contain 290 and 186 participants, respectively, are small datasets compared to the MIMIC online waveform database and University of Queensland Vital Signs datasets, which many researchers use to train their BP predictive models^18,19^. Because the size of the training dataset is known to substantially affect the performance of neural-network-based BP prediction models^20^, additional performance improvements can be expected by collecting additional training datasets using the developed sensor. However, clinical data collection is hampered by high cost, excessive time, and other limitations. Including inter- and intra-individual BP variation is important for the evaluation of cuffless devices, but difficult to obtain^21^. Our acquired dataset only acquired BP under static conditions and did not consider BP variation within each individual. Additionally, demographics (eg, age, gender) are often used as additional inputs to BP prediction models^21^. However, our proposed model does not take advantage of this to relieve the hassle of requiring users to enter demographic information.

In our study, dataset1 and dataset2 had slightly different conditions, such as the data acquisition environment, location, and some sensor specifications. As mentioned earlier, when the BP estimation system is trained using two datasets with different domains, it is difficult to expect a high accuracy in the target dataset^22^. The proposed BP estimation system was verified using five-cross-validation by setting dataset2 as the target dataset. we used regularization terms and dropout techniques to prevent model overfitting. and we showed the performance of the model through 5-fold cross validation. at this time, all performances for each 5-fold were shown. we applied the methods to prevent model overfitting with insufficient clinical data, as in other studies. If we apply a domain adaptation technique^23^ that can achieve better performance for the target dataset while reducing the interval between datasets in different domains^24^, we believe that we can obtain a more accurate estimated BP from dataset2.

According to the Association for the Advancement of Medical Instrumentation (AAMI) standard^25^, the BP estimation error should be within 5±8mmHg. The proposed BP estimation system satisfied the AAMI criterion for DBP, and the SBP was also close to the AAMI criterion. The accuracy of our proposed BP estimation system can be improved if more data are collected, and the domain mismatch in the datasets collected in different environments between datasets owing to features of different domains are resolved. We plan to improve the deep-learning-based BP estimation system to obtain more accurate predicted BP values by applying domain adaptation techniques to our two datasets with different characteristics.

## Method

We propose a novel BP prediction system with an attention mechanism that uses multi-channel PPG signals and a finger pressure signal. The proposed BP prediction system can learn to extract features from raw PPG and finger pressure signals using an end-to-end deep-learning method without relying on human-engineered, hand-crafted feature extraction methods. Moreover, an attention mechanism allows the proposed system to effectively combine features extracted from each PPG channel.

**Figure 6:**
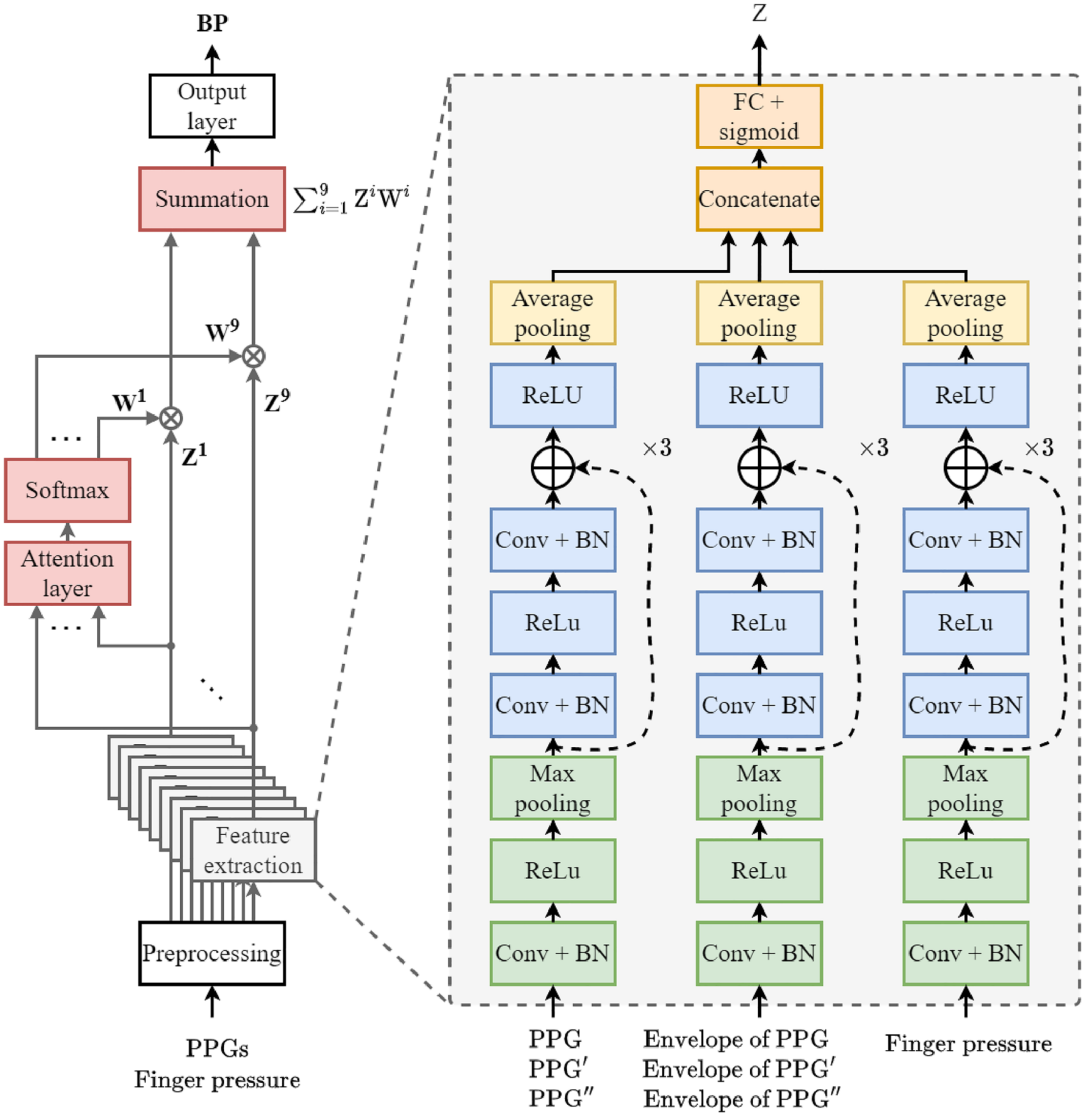
Proposed blood pressure estimation system.

**Figure 7:**
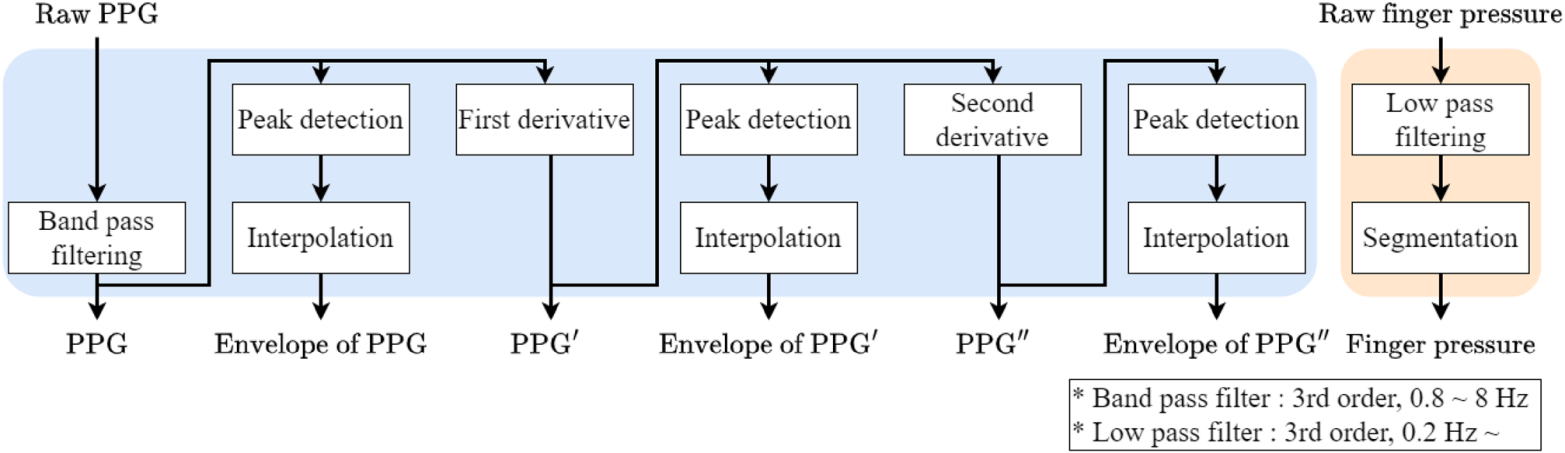
Signal preprocessing block diagram of raw PPG, and finger pressure signals.

### Signal preprocessing and data preparation

Because the acquired raw PPG and finger pressure signals contained noise components, filtering was applied to remove noise. Specifically, to use various input signals for the feature extraction model, preprocessing steps were performed to obtain the filtered PPG signal envelope and differential signals (i.e., the first- and second-order temporal derivatives). Previous studies on BP prediction using PPG signals showed that using the first- and second-order differential signals of PPG in addition to the PPG signal, the BP prediction model can more accurately predict BP by modeling various information^26,27^. Therefore, we modeled the BP prediction system by adding the envelope signal and the first- and second-order differential signals. A block diagram of the signal preprocessing method is shown in detail in Fig. 7. To remove noise components, the raw PPG signal was passed through a band-pass filter with a cut-off frequency of 0.8–8 Hz for each multi channel signal. We also obtained the PPG envelope ${X}_{e}$ from the filtered PPG signal ${X}_{p}$ to provide various types of information to the CNN-based feature extractor. The PPG envelope was calculated through peak detection of the filtered PPG signal and interpolation. After obtaining the filtered PPG and PPG envelope signals, the first- and second-order derivative signals were obtained $(\triangle {X}_{p}, \triangle ^{2}{X}_{p}, \triangle {X}_{e},$ and $\triangle ^{2}{X}_{e})$ to increase the diversity of the input as described above. The raw finger pressure signal was passed through a low-pass filter with a cut-off frequency of 0.2 Hz. We segmented the finger pressure signal from the maximum point of the PPG envelope signal to the left and right intervals of 5 seconds.

Through the signal preprocessing described above, we constructed a dataset $({X}_{1}, {X}_{2}, {X}_{3},$ and *Y*) to train the proposed BP prediction system, where ${X}_{1}(={X}_{p} \oplus \triangle {X}_{p} \oplus \triangle ^{2}{X}_{p})$ is a concatenated input of PPG-related signals with dimensions of 1720$\times$3. In addition, ${X}_{2}(={X}_{e} \oplus \triangle {X}_{e} \oplus \triangle ^{2}{X}_{e})$ is the concatenated input of PPG envelope-related signals with dimensions of 1720$\times$3, and ${X}_{3}(={X}_{f})$ is a filtered finger pressure signal with dimensions of 215$\times$1.

### CNN-based feature extraction

The CNN successfully learns the relationship between neighboring data points through a convolution operation and can compress information from the input signal through a pooling layer^28^. Thus, we constructed three parallel input streams of the CNN model such that the features were extracted for each of the ${X}_{1}$, ${X}_{2}$, and ${X}_{3}$ inputs. The overall architecture of the proposed deep learning-based BP estimation system is shown in Fig. 6. The three input streams, expressed as $C(\cdot ): X_{i} \rightarrow Z_{i}$, where *X* and *Z*, denote the input and extracted features, respectively, and *i* $\in$ $\{1, 2, 3\}$ denotes the type of input feature. Each input stream first applies a convolution, batch normalization (BN), and the ReLU activation function, followed by a max-pooling operation. Subsequently, three CNN blocks, each with two repeats of convolution, BN, and ReLU with a residual connection, are stacked, and an average pooling layer aggregates the information from each feature stream. Finally, the features extracted from the three input streams are concatenated to form a single feature $Z= {Z}_{1} \oplus {Z}_{2} \oplus {Z}_{3}$. The concatenated feature, *Z*, is then introduced to a fully connected layer with sigmoid nonlinearity, producing the final latent feature containing various types of information extracted from different input signals. Residual connections resolve the vanishing gradient problem^29^ when training the feature extraction model. After the CNN-based feature extraction models are trained for each PPG channel, the attention mechanism can be trained to combine multi channel features for BP prediction.

To train the feature extraction model, the last output layer produces the estimated BP ${\hat{y}} \in {\mathbb {R}}$. The model is then trained to minimize the mean squared error (MSE) between the reference and estimated BPs:

1 $$\begin{aligned} {\mathscr {L}}_{MSE}=\frac{1}{N}\sum _{i=1}^{N}(y_{i}-{\hat{y}}_{i})^{2}, \end{aligned}$$

where *N* denotes the number of samples; ${y}_{i}$ denotes the reference BP; and ${\hat{y}}_{i}$ denotes the estimated BP. To extract features from each of the 9-channel PPG signals, we trained nine feature extraction models for each PPG channel. The trained 9-channel feature extraction models can be expressed as $f(\cdot ) : [{X}^{i}_{1}, {X}^{i}_{2}, {X}^{i}_{3}] \rightarrow Z_{i}$, where *Z* is the final latent feature, and *i* $\in \{1,2,...,9\}$ is the PPG channel index. Nine latent features ${Z} \in {\mathbb {R}}^{16\times 9}$ with 16 dimensions were used as inputs to the multi channel attention model for the final BP prediction. The attention-based multi channel BP estimation performance is compared with the per-channel BP estimation performance in Section IV.

### Attention mechanism

Recently, attention mechanisms have proven effective in many fields, such as speech recognition^30,31^ and natural language processing^32^. Attention mechanisms are neural networks that focus on important regions. We extracted each feature from a CNN-based feature extraction model using multi channel PPG and finger pressure signals. However, because the position of the finger placed on the proposed multi channel PPG sensor and the characteristics of the fingers may be different for each user, the importance of each channel for BP estimation may also differ for each user. Therefore, we applied an attention mechanism to the proposed BP estimation system for adaptively weighing channel-wise features according to their importance in estimating the BP for each user.

As shown in Fig. 6, the extracted features, ${Z}^{i}$, for the PPG channels, ${i} \in \{1, \ldots , 9\}$, were introduced to the attention layer comprising a single-layer perceptron, ${s}(\cdot ) : {Z}^{i} \rightarrow {S}^{i}$, to obtain a score, ${S}^{i}$ representing the importance of each channel. Score, ${S}^{i}$, is obtained as follows:

2 $$\begin{aligned} S^{i}=s(\omega Z^{i}+b), \end{aligned}$$

where $\omega$ and *b* are the trainable weights and biases, respectively. Subsequently, from the obtained score, ${S}^{i}$, the attention weight, ${W}^{i}$, was calculated using the softmax function to indicate the importance of each channel as a probability value. the attention weight, ${W}^{i}$, was calculated as follows:

3 $$\begin{aligned} W^{i}=\text {softmax}(S^{i})=\frac{\text {exp}(S^{i})}{\sum _{i}{}{\text {exp}(S^{i})}}. \end{aligned}$$

The attention-weighted feature, ${Z}^{\prime } \in {\mathbb {R}}^{16\times 1}$, was obtained through the weighted summation of the attention weight, ${W}^{i}$, and the corresponding feature, ${Z}^{i}$. The proposed BP estimation system produces an estimated BP through the output layer using the attention-weighted feature, ${Z}^{\prime }$. To train the attention mechanism model, the MSE loss was calculated between the reference and estimated BPs ${\hat{y}}$ obtained from the output layer of the attention mechanism. The proposed model was trained separately to minimize the MSE for systolic and diastolic BP.

## Conclusion

In this study, we developed a multi-channel PPG sensor that acquires multi-channel PPG signals at different wavelengths. Moreover, we devised a deep-learning-based BP estimation system that can predict BP from multi-channel PPG signals acquired from the proposed sensor and finger pressure signal. The proposed BP estimation system can extract features without human engineering and accurately predict the BP through an attention mechanism. Through attention weight analysis, we confirmed that the attention mechanism can improve the prediction performance of hypertension and hypotension. Because the proposed deep-learning-based BP estimation system is a cuff-free and calibration-free method, it is possible to monitor BP regularly and has the potential to diagnose hypertension at an early stage. The proposed BP estimation system can potentially enable regular BP monitoring of multiple users through mobile devices, such as smartphones or smart wristwatches.

## Data Availability

The data that support the findings of this study are available via e-mail from the corresponding author upon reasonable request.
